# Mechanism of Dinitrochlorobenzene-Induced Dermatitis in Mice: Role of Specific Antibodies in Pathogenesis

**DOI:** 10.1371/journal.pone.0007703

**Published:** 2009-11-05

**Authors:** Elizabeth Yan Zhang, Aaron Yun Chen, Bao Ting Zhu

**Affiliations:** Department of Pharmacology, Toxicology and Therapeutics, School of Medicine, University of Kansas Medical Center, Kansas City, Kansas, United States of America; Institut Pasteur, France

## Abstract

**Background:**

Dinitrochlorobenzene-induced contact hypersensitivity is widely considered as a cell-mediated rather than antibody-mediated immune response. At present, very little is known about the role of antigen-specific antibodies and B cells in the development of dinitrochlorobenzene-induced hypersensitivity reactions, and this is the subject of the present investigation.

**Methodology/Principal Findings:**

Data obtained from multiple lines of experiments unequivocally showed that the formation of dinitrochlorobenzene-specific Abs played an important role in the development of dinitrochlorobenzene-induced contact hypersensitivity. The appearance of dinitrochlorobenzene-induced skin dermatitis matched in timing the appearance of the circulating dinitrochlorobenzene-specific antibodies. Adoptive transfer of sera containing dinitrochlorobenzene-specific antibodies from dinitrochlorobenzene-treated mice elicited a much stronger hypersensitivity reaction than the adoptive transfer of lymphocytes from the same donors. Moreover, dinitrochlorobenzene-induced contact hypersensitivity was strongly suppressed in B cell-deficient mice with no DNCB-specific antibodies. It was also observed that treatment of animals with dinitrochlorobenzene polarized Th cells into Th2 differentiation by increasing the production of Th2 cytokines while decreasing the production of Th1 cytokines.

**Conclusions/Significance:**

In striking contrast to the long-held belief that dinitrochlorobenzene-induced contact hypersensitivity is a cell-mediated immune response, the results of our present study demonstrated that the production of dinitrochlorobenzene-specific antibodies by activated B cells played an indispensible role in the pathogenesis of dinitrochlorobenzene-induced CHS. These findings may provide new possibilities in the treatment of human contact hypersensitivity conditions.

## Introduction

Dinitrochlorobenzene (DNCB)-induced contact hypersensitivity (CHS) of the skin in mice is a commonly-used animal model for studying the pathogenesis of contact dermatitis [1]. Mechanistically, it is generally thought that upon topical application, DNCB or other chemicals with a similar structure, such as 2,4-dinitrofluorobenzene and picryl chloride, can complex with various skin proteins to form covalent conjugates and thereby function as immunogen(s). DNCB-modified macromolecules are then internalized by local APCs, such as skin Langerhans cells, dermal dendritic cells and macrophages, processed and presented to T cells for activation [2], [3]. Approximately twenty-four hours after subsequent exposures to DNCB (often referred to as “challenges”), macrophages were found to accumulate at the site of DNCB exposure [4], contributing to the development of dermatitis (e.g., ear swelling and skin inflammation).

At present, the prevailing explanation is that the pathogenesis of DNCB-induced CHS is predominantly the result of T cell-mediated immune responses [4]–[7]. However, there were also studies in recent years suggesting the possible involvement of B cells in the development of CHS. It was recently reported that animals exposed to low molecular weight chemical allergens, such as 2,4-dinitrofluorobenzene, picryl chloride and oxazolone, had an increased B cell population in their draining lymph nodes [8]. Similarly, exposure of animals to contact allergens also led to an increased percentage of antigen-specific B-1 cells (which mostly produce IgM) in the draining lymph nodes [9]–[11]. Besides these scattered pieces of data, however, little is known about the roles of Abs and B cells in the development of DNCB-induced hypersensitivity reactions.

In the present study, we have sought to explore the pathogenic role of DNCB-specific Abs in the development of CHS in a mouse model. We have gathered several lines of experimental evidence showing that the formation of DNCB-specific Abs plays an important role in the development of DNCB-induced CHS.

## Results

### Induction of Skin CHS in Mice by DNCB

Induction of ear swelling by topical application of DNCB (or other similar chemicals) in mice has been widely used for decades as an animal model for studying the pathogenesis of CHS. Typically, the animals are first sensitized once by painting the chemical on their shaved back or abdominal skin. Five days later, they are challenged on one of the ears. The thickness of the ear is measured 24 h later to determine the level of swelling [12], [13]. In the present study, we have modified the DNCB treatment scheme by giving multiple sensitizations with adequate time intervals. We found that different schemes of DNCB administration produced variable degrees of ear swelling (data are summarized in **Table S1**). In general, factors including gender, DNCB dosage (*i.e.*, the concentration of its solution and the volume applied), solvent and method of administration, did not appear to significantly affect the severity of DNCB-induced ear swelling. However, the number of DNCB sensitizations and particularly the duration between the sensitizations were found to be the most important factors affecting the severity of ear swelling. If the interval between the first and second DNCB sensitizations was sufficiently long (7 days and up), strong ear swelling was readily observed. Otherwise, the severity of DNCB-induced ear swelling in the animals would be low (<50%) and very inconsistent (**Table S1**).

**Figure 1:**
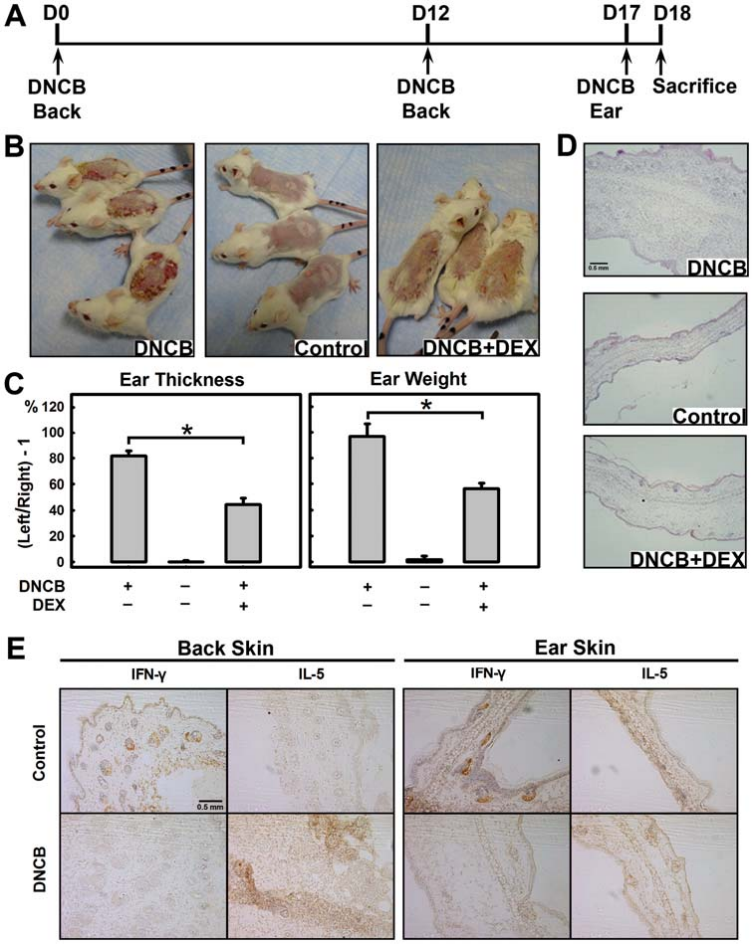
Induction of CHS by topical administration of DNCB and its suppression by DEX. **A.** The DNCB treatment schedule used in this experiment. As shown, the mice were first painted with 100 µL of 2% DNCB or vehicle at their shaved back on D0 as the first sensitization. On D12, mice received the second sensitization with DNCB in the same way as the first one. On D17, mice were challenged with 20 µL of 2% DNCB or ETOH (the vehicle) painted on their left ears twice with a 60-min interval. Twenty-four h later (on D18), the ear swelling was evaluated by measuring the thickness and weight. Other assays were also performed after the animals were sacrificed. DEX was administrated through i.m. injection at 10 mg/kg b.w. every other day starting from a day before the first sensitization until the end of the experiment. **B.** The severity of back skin inflammation after sensitization with DNCB. The photos were taken at 8 days after sensitization with DNCB. **C.** The ear swelling indices (ear thickness and ear weight). They were calculated according to the differences between the DNCB-challenged left ears and the unchallenged right ears using the following formula: index = the measured value for the left ear/the measured value for the right ear - 1. * *P*<0.01 compared to animals treated with DNCB alone. Each value is the mean±S.D. (N = 6). **D.** Histological changes in the ears (H/E staining). **E.** Comparison of IFNγ and IL-5 immunohistochemical staining in the back and ear skins of mice treated with DNCB or vehicle. The back skin and the left ear tissues were harvested on D18 (one day following DNCB challenge of the ear). The skin sections were stained with anti-mouse IFNγ or anti-mouse IL-5, respectively. Six animals per group were used, and a representative slide for each group is shown.

Using an optimized DNCB sensitization scheme (**Figure 1A**), we then characterized the pathogenic changes in the back and ear skins. We found that all animals developed strong skin hypersensitivity reactions in the painted back skin after ≥8 days of initial DNCB sensitization (**Figure 1B**). The scaring was severe and lasted for a couple of days. Similarly, at 24 h after DNCB challenge of the ear (note that the ear had no prior exposure to DNCB), significant ear swelling was also readily observed in animals previously exposed to DNCB compared to control animals (**Figure 1C**). The ear swelling following DNCB challenge was not due to a non-specific inflammatory response to the chemical (**Figure S1**).

Histopathological analysis showed that pathological changes of both back and ear skins were mainly located within the dermis and characterized by the presence of inflammatory infiltration, vascular congestion, and moderate edema (representative slides of ear skin tissue are shown in **Figure 1D**). Administration of dexamethasone (DEX) (at 10 mg/kg b.w., i.m.) once every two days during DNCB treatment markedly reduced back skin inflammation (**Figure 1B**) and also ear swelling (**Figure 1C, 1D**).

Immunohistochemical analysis showed that DNCB-painted mice had more IL-5-positive cells but fewer IFN-γ-positive cells in their back skin compared to vehicle-treated animals (**Figure 1E**). Similarly, more IL-5-positive cells but less IFN-γ-positive cells were observed in the ear tissue one day after DNCB challenge compared to vehicle-treated animals (**Figure 1E**). Administration of DEX was accompanied by a marked decrease in IL-5-positive cells in the ear tissue (data not shown). Collectively, these results suggest a predominance of Th2 cytokine-mediated inflammatory response at the sites of DNCB exposure.

In these experiments, the body and organ weight changes were also determined (summarized in **Figure S2**). Weight index for each organ was calculated according to the following formula: weight index = organ wet weight (mg)/body weight (g). We found that treatment with DNCB did not markedly alter the body weight or spleen weight index, but did decrease the thymus weight index by ∼30% compared to vehicle-treated animals. Treatment with DNCB plus DEX decreased the animal body weight by 11%, spleen weight index by 60%, and thymus weight index by 80% compared to DNCB treatment alone.

### Role of DNCB-Specific Abs in the Induction of CHS

To probe the role of DNCB-specific Abs in the induction of CHS, we determined whether the onset of CHS coincided with the appearance of DNCB-specific Abs, and also whether the severity of CHS correlated with serum levels of DNCB-specific Abs.

**Figure 2:**
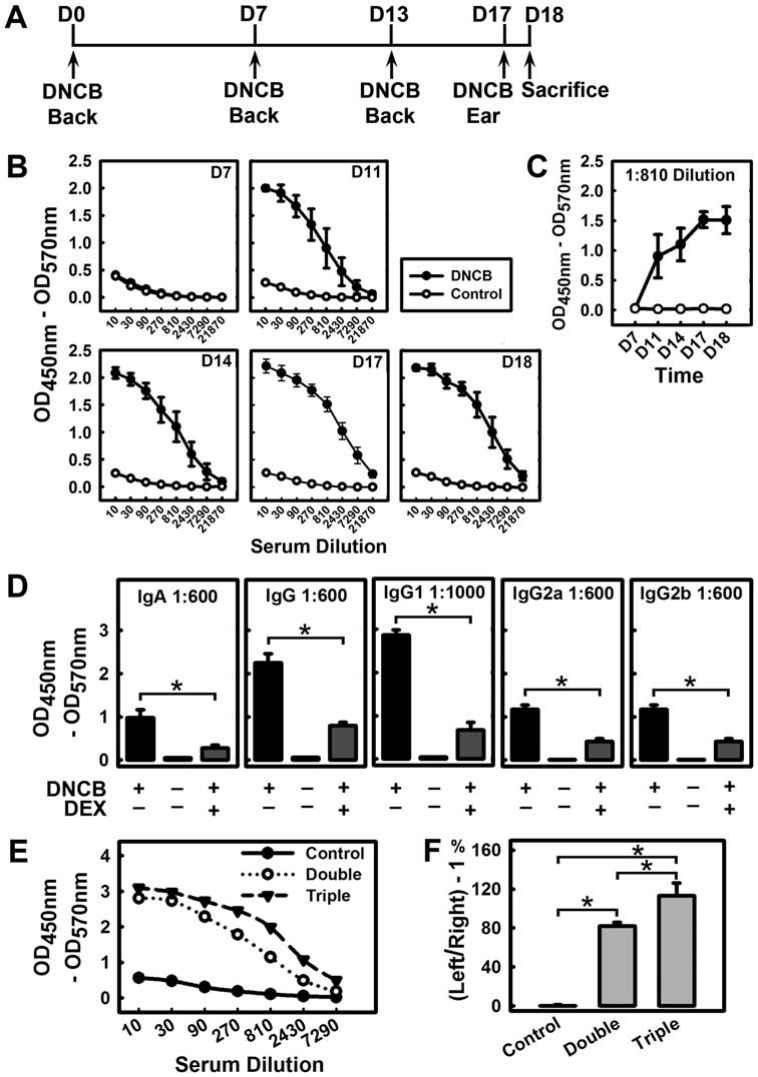
Role of DNCB-specific Abs in the induction of CHS. **A.** The DNCB treatment schedule used in this experiment. On D0, mice were sensitized by painting DNCB or vehicle on their shaved backs, respectively. On D7 and D13, mice were painted with DNCB or vehicle on their shaved back again in the same way as the first sensitization. On D17, mice were challenged with painting DNCB or vehicle, respectively, on their left ears. Mice received a total of three back paintings and one ear challenge, and the experiment was terminated on D18. **B and C.** Serum levels of DNCB-specific IgG Abs on different days following DNCB treatment. The blood samples were collected on the following days: D7 (right before the second sensitization), D11 (between the second and third sensitizations), D14 (after the third sensitization), D17 (right before DNCB ear challenge), and D18 (the animals were sacrificed at 24 h after DNCB challenge). Each value is the mean±S.D. (N = 4). **D.** Comparison of DNCB-specific IgA, IgG, IgG1, IgG2a, and IgG2b Ab levels in different treatment groups. Each value is the mean±S.D. (N = 6). **E.** Serum DNCB-specific IgG Abs on D18. The double DNCB sensitizations before challenge were applied on D0 and D12 as described in **Figure 1A**, whereas the triple sensitizations were applied on D0, D7 and D13 as depicted in **Figure 2A**. Both groups were received DNCB challenge on D17 and terminated a day later. One representative curve from each group (N = 4) is shown. **F.** Ear swelling as measured according to the thickness of left/right ear - 1. * *P*<0.01 compared to the DNCB treatment group. Each value is the mean±S.D. (N = 4).

We found that treatment of animals with DNCB (**Figure 2A**) increased serum levels of DNCB-specific Abs, and this effect largely depended on the duration and frequency of DNCB treatments. No appreciable levels of DNCB-specific Abs were found in sera of DNCB-treated mice until at least 8 days after the first DNCB sensitization (**Figure 2B, 2C**). Various subtypes of DNCB-specific Abs, including IgG, IgG1, IgG2a, IgG2b and IgA (but not IgE), were detected in the serum of these animals at ≥8 days following the initial DNCB sensitization(s) (**Figure 2D** and **Figure S3**). In addition, triple sensitizations with DNCB induced higher titers of DNCB-specific Abs compared to double sensitizations (a representative data set is shown in **Figure 2E**). Treatment of these animals with DEX decreased by over 50% the serum levels of DNCB-specific Abs, including IgA, IgG, and its subtypes, IgG1, IgG2a and IgG2b (**Figure 2D** and **Figure S3**). For comparison, we have also determined the total serum Ab levels (summarized in **Figure S4**). DNCB treatment did not change the serum levels of total IgM, IgG2a, IgG2b and IgG3, but it slightly increased the serum levels of total IgA, IgG and IgG1. The effect of DEX on the total serum Ab levels was very mild.

By comparing the appearance of DNCB-specific Abs with the occurrence of skin inflammation, we found that before DNCB-specific Abs became detectable in serum, no appreciable signs of skin reactions at the site of DNCB sensitization could be observed. Once the skin inflammation started to appear in the DNCB-treated animals (**Figure 1B**), large quantities of DNCB-specific Abs were always concomitantly detected in their sera (representative data for IgG are shown in **Figure 2B, 2C**). In general, a higher degree of severity was accompanied by the presence of higher levels of DNCB-specific Abs in serum. For instance, triple sensitizations with DNCB induced stronger back skin inflammation compared to double or single sensitization(s), which agreed with the change in the levels of DNCB-specific serum Abs (**Figure 2E**). Similarly, treatment with DEX reduced the back skin inflammation, to an extent that was comparable to its reduction of the serum levels of DNCB-specific Abs (**Figure 2D** and **Figure S3**).

Similarly, the severity of ear swelling following DNCB challenge also closely matched the serum levels of DNCB-specific Abs. We observed that when the animals had no detectable serum levels of DNCB-specific Abs, their ear swelling was always mild, and the parameter of Δ ear thickness (*i.e.*, thickness of the DNCB-challenged ear/the control ear - 1), was almost always below 0.5, and severe ear swelling was always observed along with high titers of serum DNCB-specific Abs, particularly in the cases of triple sensitizations (**Table S1**, compare Exp-1 through Exp-9 with Exp-10 and Exp-11). As shown in **Figure 2F** (data from a representative experiment), the ear thickness in mice topically sensitized twice with DNCB was increased by 82% at 24 h after DNCB challenge compared to control animals, whereas the ear thickness in mice with triple sensitizations was increased by 113%.

### Induction of CHS by Adoptive Transfer of DNCB Antisera

**Figure 3:**
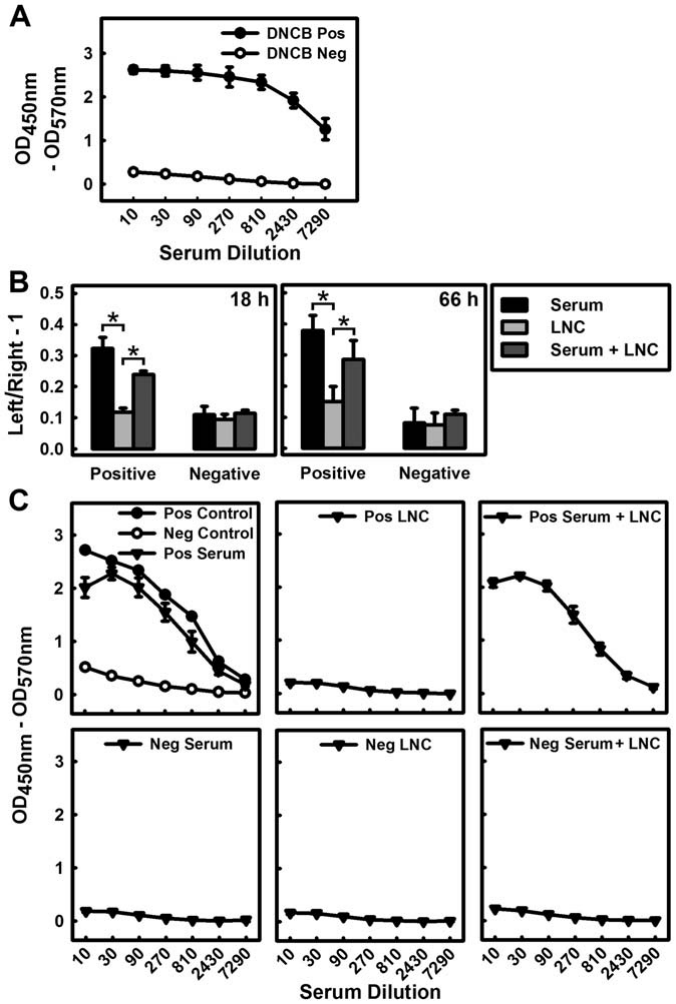
Effect of adoptive transfer of sera and/or lymphocytes on the development of DNCB-induced CHS. **A.** DNCB-specific IgG Abs in the sera of donor mice. The DNCB-positive donors (close circle •) were exposed to DNCB three or four times with sufficient time intervals and the DNCB-negative donors (open circle ○) were only treated with vehicle. **B.** Ear swelling of recipient mice from different treatment groups. After *i.v.* injection of serum only, LN lymphocytes (LNC) only, or combination of serum + LNC from either DNCB-positive donors (Pos) or DNCB-negative donors (Neg), the recipient animals in these 6 different treatment groups were challenged on their left ears with DNCB and then their ear swelling was measured 18 h (left panel) and 66 h (right panel) later. **C.** DNCB-specific IgG Abs in the sera of recipient animals from the 6 treatment groups. The levels were determined at 66 h after DNCB ear challenge. * *P*<0.01 compared to the positive LNC group (mice adoptively transferred with DNCB-positive lymphocytes). Each value is the mean±S.D. (N = 5 for each of the three Pos groups, and N = 3 for each of the three Neg groups).

To provide definitive evidence that DNCB-specific Abs played an essential role in the development of skin hypersensitivity reactions, we conducted experiments by transferring sera collected from DNCB-sensitized mice to determine whether the presence of DNCB-specific Abs alone could transfer similar hypersensitivity skin reactions to recipient animals (with no prior exposure to DNCB) when they were challenged with DNCB. First, the donor animals were treated multiple times with DNCB to induce strong CHS, and their sera were tested to ascertain that high titers of DNCB-specific Abs were produced before they were used for adoptive transfer into recipient animals. Meanwhile, sera from mice that were not exposed to DNCB were used as control (**Figure 3A**). In addition, for the purpose of comparison, lymphocytes from lymph nodes were also prepared from the same animals (DNCB-treated and control animals) and used for adoptive transfer.

Eighteen and 66 h after the adoptive transfer of sera containing high titers of DNCB-specific Abs into the mice, DNCB challenge of their ears produced a markedly stronger ear swelling compared to animals that received the DNCB-negative sera (**Figure 3B**). The recipient animals that received DNCB antisera produced a sustained ear swelling after DNCB challenge on their left ears even at 66 h after the transfer. Also, in these animals, the presence of DNCB-specific Abs in their sera was readily detected (**Figure 3C**). Notably, the ear swelling in animals receiving the DNCB-positive lymphocytes was not significantly different from animals that received the DNCB-negative sera or lymphocytes or sera plus lymphocytes. The recipients that were adoptively transferred with DNCB-positive sera plus DNCB-positive lymphocytes produced a reduced level of ear swelling after DNCB challenge compared to animals receiving the DNCB-positive sera alone (tested at up to 66 h post-transfer) (**Figure 3B**). Overall, the DNCB-specific IgG levels in the sera of recipients were closely associated with the severity of ear swelling under different adoptive transfer conditions, with the following rank order (from high to low): recipients of the DNCB-positive sera only > recipients of the DNCB-positive sera plus lymphocytes ≫ recipients of DNCB-positive lymphocytes only ≈ recipients of DNCB-negative sera only ≈ recipients of DNCB-negative lymphocytes ≈ recipients of DNCB-negative sera plus lymphocytes (**Figure 3C**).

### Role of B Cells in DNCB-Induced CHS

**Figure 4:**
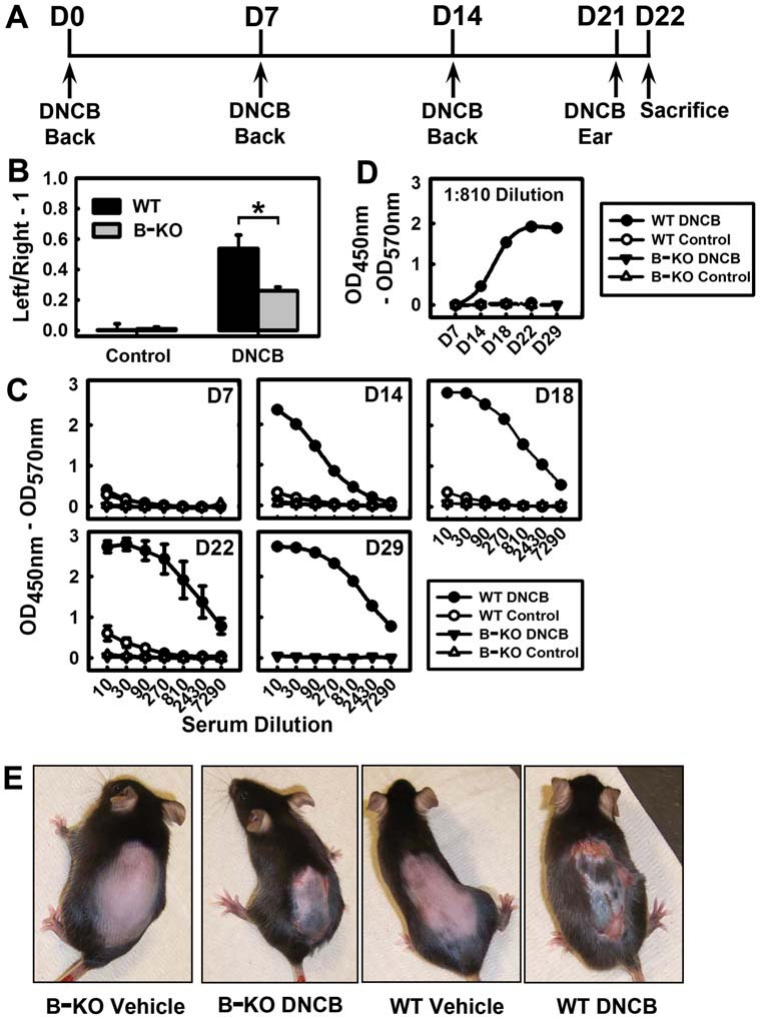
Comparison of the severity of CHS in B cell-deficient mice and control WT mice. **A.** The DNCB treatment schedule used in this experiment. The mice were sensitized on their shaved back on D0. On D7 and D14, mice were received the second and third sensitization with DNCB on their shaved back the same as the first sensitization. They were challenged on their left ears on D21, and sacrificed on D22. **B.** Ear swelling index in B-KO mice and WT mice. The animals were separated into 4 groups: WT-DNCB, WT-control, B-KO-DNCB, B-KO-control. **C and D.** Time-dependent changes of serum DNCB-specific IgG Abs in B-KO mice and WT mice. Serum Abs were determined on D7, D14, D18, D22 and D29 (note that one of the mice was sacrificed on D29 whereas all other mice were sacrificed on D22). **E.** Comparison of the back skin dermatitis between B-KO mice and WT mice. The photos show the degree of inflammatory responses in the shaved back skin of animals following second sensitization with DNCB or vehicle. * *P*<0.01 compared to WT-DNCB mice. Each value is the mean±S.D. (N = 3 for the vehicle treatment groups and N = 6 for the DNCB treatment groups).

To provide additional support for the suggested role of DNCB-specific Abs in the development of CHS, we also conducted studies using B cell-deficient (B-KO) mice. The B-KO mice and their background control (*i.e.*, the WT C57BL/6J mice) were treated with DNCB or vehicle as described in **Figure 4A**, and their ear swelling was measured at 24 h after DNCB challenge. While the WT mice had a 54% increase in ear thickness, the B-KO mice showed a significantly lower level of ear swelling (26% increase, see **Figure 4B**). Notably, we also observed that the DNCB-specific serum IgG levels in the B-KO mice remained at the baseline levels following multiple DNCB treatments (**Figure 4C**), which demonstrated that the B-KO mice could not produce antigen-specific Abs. However, the WT mice had an increased DNCB-specific serum IgG level after the second sensitization, which occurred after D7 and reached plateau around D22 (**Figure 4C, 4D**). Also, after the second sensitization with DNCB on their shaved back skin, there was a more pronounced skin dermatitis appearing on the backs of the WT mice than on the backs of the B-KO mice (**Figure 4E**).

### DNCB Treatment Promotes Differentiation of Th Cells into Th2 Cells

In the present study, we also probed the changes of cytokine profiles as well as lymphocyte compositions in the lymph nodes to determine whether there was a distinct immunological shift in the Th cell functions following DNCB treatments. We found that DNCB treatments promoted the differentiation of Th cells into Th2 cells, which appeared to provide a mechanistic basis for the predominant induction of DNCB-specific Abs as an important pathogenic change underlying the development of CHS. Some of the findings are summarized below.

#### Changes in cytokine profiles

**Figure 5:**
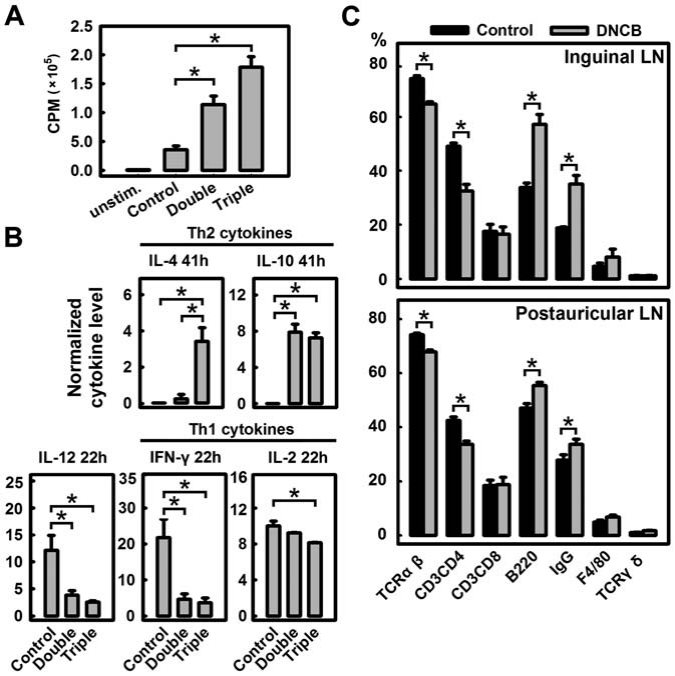
Skewing of Th cells in the Th2 direction following DNCB treatment. **A** and **B.** Double DNCB sensitizations before challenge were applied on D0 and D12 as described in **Figure 1A** whereas triple sensitizations were applied on D0, D7 and D13 as in **Figure 2A**. Both groups received challenge on D17 and terminated a day later. **A.** Proliferation of lymphocytes as measured by using the [^3^H]thymidine incorporation assay. Lymphocytes were harvested from collected LNs and cultured *in vitro* under the stimulation of Con A at 5 µg/mL for 60 h. [^3^H]Thymidine was introduced for the final 14 h and the radioactivity (cpm) was measured using a Beta counter. * *P*<0.01 compared to the control group. Each value is the mean±S.D. (N = 4). **B.** Cytokine production by cultured lymphocytes. The supernatant from cultured lymphocytes was collected at 22 and 41 h, and assayed using ELISA for cytokine concentrations. The cytokine production levels were normalized according to the relative cell density (based on the radioactivity values from the cell proliferation assay). Each value is the mean±S.D. (N = 3). **C.** The population of various cell types (TCRβ^+^, CD3^+^CD4^+^, CD3^+^CD8^+^, B220^+^, IgG^+^, F4/80^+^, and TCRγδ^+^) in the inguinal and postauricular lymph nodes of control and DNCB-treated mice. In this experiment, the DNCB treatment regimen was the same as depicted in **Figure 1A**. * *P*<0.01 compared to the control group. Each value is the mean±S.D. (N = 6).

After *in vitro* culture for 60 h, the primary lymphocytes collected from lymph nodes (LNs) of mice that received triple DNCB sensitizations, proliferated four times faster than cells from control mice that received vehicle during sensitizations and challenge. Lymphocytes in LNs from mice with double sensitizations proliferated approximately three times as fast as cells from the control animals (**Figure 5A**).

Several cytokines, including IL-2, IL-4, IL-10, IL-12 (p70) and IFN-γ, were detected in the culture medium of LN lymphocytes at different time points (22 and 41 h). Multiple DNCB sensitizations proportionally decreased the IL-12, which is secreted by macrophages to induce Th1 cell development. Multiple DNCB treatments also weakened the production of Th1 cytokines such as IL-2 and IFN-γ, while it increased the production of Th2 cytokines such as IL-4 and IL-10 (**Figure 5B**).

#### Changes in lymphocyte populations

In both postauricular LNs (the predominantly local draining LNs) and inguinal LNs (the systemic non-draining LNs), treatment of animals with DNCB consistently decreased the populations of TCRαβ^+^ and CD3^+^CD4^+^ T cells (mostly Th cells), but increased the populations of B220^+^ and IgG^+^ cells (mostly B cells) as well as the population of F4/80^+^ macrophages (**Figure 5** and **Figure S5**). In comparison, it had no appreciable effect on TCRγδ^+^ cells. DNCB also did not differentially affect the percentages of different sub-populations of lymphocytes (such as TCRαβ^+^, CD3^+^CD4^+^ T cells, B220^+^ and IgG^+^ B cells) in the local draining postauricular LNs versus the systemic non-draining inguinal LNs.

## Discussion

At present, the prevailing explanation is that the DNCB-induced CHS is a condition predominantly mediated by cellular immune responses [4]–[7]. In partial support of this hypothesis, earlier studies reported that the severity of CHS was significantly lessened in mice lacking either CD4^+^ or CD8^+^ T cells, and these data suggested that the involvement of T cells in the development of CHS [7]. A recent study demonstrated that galectin-1 could induce significant cell death of Th1 and Tc1 cells but did not exert an appreciable inhibition of CHS [14], thus suggesting that CHS is largely not mediated by Th cells. In the present study, we have gathered several lines of experimental evidence (discussed below) which definitively show that the formation of DNCB-specific Abs plays an important role in the development of DNCB-induced CHS.

Firstly, we observed that before visible signs of DNCB-induced inflammatory reactions in the back skin (the site of DNCB application) could be observed, serum DNCB-specific Abs were usually below the detection limit. However, once large quantities of DNCB-specific Abs became detectable in sera, inflammatory reactions at the back skin also started to appear. The titers of DNCB-specific Abs were higher in animals sensitized multiple times with DNCB compared to animals receiving a single DNCB sensitization, and likewise, the severity of dermatitis at the site of DNCB application was always higher in animals with multiple DNCB sensitizations as opposed to single sensitization. Similar observations were also made with ear swelling following DNCB challenge. The concurrence of CHS and DNCB-specific Abs was not only observed in the BALB/c mice, a strain that is usually considered to be Th2-skewing [15], [16], but it was also observed in the C57BL/6 mouse strain, which tends to be Th1-shewing [15]. In addition, we observed that treatment of animals with DEX strongly mitigated DNCB-induced CHS in both their back and ear skins, and this inhibitory effect of DEX correlated closely with the reduction of serum levels of DNCB-specific Abs (by ∼50%) and also IL-5-positive cells in the local hypersensitive sites.

Notably, we observed that in those animals that were sensitized with DNCB only once and did not receive DNCB challenge later, relatively mild skin hypersensitivity reactions were also developed at the site of sensitization starting about eight days later. We believe this phenomenon was not in contradiction with the suggested causal role of DNCB-specific Abs in the development of hypersensitivity reactions. The fact was that once DNCB was painted on the mouse back skin, a fraction of the chemical would remain on the back skin, as evidenced by the characteristic light yellow color of this chemical remaining on their back skins well after 8 days. When the DNCB-specific Abs were formed and started to circulate in large quantities in the body (usually at 8 days after the initial DNCB sensitization), the residual DNCB on the mouse back skin would serve as a weak challenge, resulting in the development of hypersensitivity reactions of the same type as seen in those animals that were challenged with DNCB, although the intensity, as expected, would be relatively milder. Notably, before the antibodies became detectable, no hypersensitivity reactions were seen in these animals. We believe the precise timing between the appearance of hypersensitivity reactions and the appearance of specific Abs, in fact, offers additional support for the importance of specific Abs in the pathogenic process.

Secondly, we found that the adoptive transfer of DNCB-specific antisera produced the strongest response to DNCB challenge in the skins of recipient animals compared to the adoptive transfer of lymphocytes or antisera + lymphocytes. Notably, in some of the earlier studies [7], [12], adoptive transfer of lymphocytes followed by challenge with the same hapten was used to induce CHS in recipient animals. Under these experimental conditions, the ear swelling seen in the recipient animals was always milder in severity compared to animals sensitized directly with an allergen. However, the observation made in the present study was distinctly different in that the animals receiving the DNCB antisera had nearly the same intensity of hypersensitivity skin reactions when challenged with DNCB (note that these animals had no prior DNCB exposure). This distinction also points to the importance of the DNCB-specific Abs in the pathogenesis of skin hypersensitivity reactions.

Here it should also be noted that while the blood levels of DNCB-specific Abs in animals that were adoptively transferred with DNCB antisera alone or antisera plus LN cells (both obtained from DNCB-positive donors) were comparable, the ear swelling in the latter was decreased when it was compared with the former. While the mechanism of this suppressive effect exerted by LN cells is not understood at present, it is speculated that some of the components such as the suppressive regulatory T cells and/or other inhibitory factors contained in the LN might exert an inhibitory effect on the overall immune/inflammatory responses.

Thirdly, we found that the B cell-deficient mice (lacking mature B cells and also the hapten-specific Abs) had markedly lower hypersensitivity in their skin reactions and ear swelling compared to the WT mice when the same DNCB treatments were given to mice of both genotypes. This observation provides definitive evidence for the critical role of B cells and hapten-specific Abs in the pathogenic process. However, it should also be noted that DNCB could still induce, albeit to a much milder degree, inflammatory skin reactions in the B-KO mice, which suggests that other cell types, such as natural killer cells [13], may also contribute to the pathogenic process, in addition to B cells and their specific Abs.

In the present study, we have probed the changes in cytokine profiles as well as lymphocyte compositions in the lymph nodes to determine whether there is a distinct shift in the lymphocyte functions following DNCB treatment. We found that DNCB effectively induced the differentiation of T cells in the Th2 direction, which appears to provide a mechanistic basis for the predominant induction of DNCB-specific Abs and their importance in the development of CHS. Our *ex vivo* experiments showed that DNCB treatment *in vivo* increased Con A-stimulated release of IL-4 and IL-10 but decreased Con A-stimulated release of IFN-γ, IL-2 and IL-12 (p70), and these effects were more pronounced when multiple *in vivo* DNCB treatments were employed. In addition, at the sites of hypersensitivity reaction (*i.e.*, the painted back skin and painted left ears), DNCB also increased the IL-5- positive cells but decreased the IFNγ-positive cells after multiple applications of DNCB. Consistent with our observation of a Th2 shift following treatment with DNCB, earlier studies have suggested that the IL-4-producing T cells may be involved in the development of CHS [9], [11], [17]–[19]. It was also reported that while IL-4 expression was increased in the lymph nodes, IL-5 expression was significantly elevated in the ear of animals topically treated with 2,4-dinitrofluorobenzene (an analog of DNCB) [20]. Moreover, the severity of CHS was higher in IL-5 transgenic mice that had a much higher level of IL-5 expression [21], whereas the severity was markedly diminished in IL-4 deficient mice [18], [22] or in mice deficient in STAT6 (a mediator of IL-4's biological actions) [23]. Similarly, the results of the present study also showed that the degree of ear swelling seen in the Th2-skewing BALB/c mice treated with DNCB was significantly stronger than what was seen in the Th1-skewing C57BL/6 mice receiving the same DNCB treatment (**Figure 2F** **and** **Figure 4B**). Collectively, these observations provide additional indirect evidence for the mechanistic explanation proposed.

The results of our present study showed that administration of DNCB induced the formation of DNCB-specific IgG1 to a greater extent than other Ig subtypes. This observation is consistent with our observation that DNCB treatments induced Th2-skewing since Th2 cells are known to play an important role in mediating the secretion of antigen-specific IgG1 from B cells and IL-4 is known to induce isotype switching favoring IgG1 synthesis [24], [25]. It is speculated that when the anti-DNCB IgG1 antibodies start to be formed in large quantities and circulate in the body, they would conjugate with the antigen molecules (applied during challenge) to form immune complex deposits, which would subsequently activate the complement system and thereby contribute to the development of hypersensitivity reactions. This mechanistic element is usually characteristic of many type III hypersensitivity reactions [26], [27]. Although DNCB-induced CHS has been mostly considered a type IV hypersensitivity reaction, the findings of our present study suggest that this condition may share some of the immunological characteristics of both types III and IV reactions. More studies are needed to further test this hypothesis.

The observations made in the present study in the mouse model agreed well with some of the earlier observations made in patients with contact dermatitis. It was reported earlier that there was an increase in the expression of IL-4 and IL-5 mRNAs following allergen challenge [19], [28], together with an augmented Th2 response and a diminished Th1 response [29]. Similarly, the IL-4 producing cells in patients with dermatitis were increased in the CD4^+^ and CD8^+^ T cell population among the peripheral blood mononucleocytes [30]. Moreover, patients with contact dermatitis had a higher percentage of peripheral blood lymphocytes expressing Ig proteins when compared to normal subjects [31], [32], and repeated elicitations with antigen altered the balance of cytokines released locally, with a shift toward Th2-dominated responses [33]. All these observations are in accord with our data showing a crucial role of Th2 cytokines and allergen-specific Abs in the development of CHS. The human relevance of these observations is expected to aid in the development of novel approaches for treating human CHS conditions.

Lastly, it is of note that the skin CHS developed in rodents following topical application of DNCB or other similar chemicals has been used for decades by many researchers as a model to study T cell-mediated hypersensitivity immune responses. At present, the most commonly-adopted protocol includes the use of one DNCB sensitization followed by one DNCB challenge 5 days later. However, if the chemically-induced CHS is, in fact, mediated predominantly by B cells and their specific Abs, the use of this animal model may not serve the purposes as originally intended. Based on the systematic comparison of various treatment methods made in the present study, we also noticed that different methods of DNCB administration could produce very different levels of inflammatory ear swelling (**Table S1**). In general, factors like gender, DNCB dosage, vehicles, and method of administration appeared to be of little importance in the induction of ear swelling, but the number of DNCB treatments and particularly the time intervals between the treatments were found to be the most crucial factors that affected the severity of ear swelling. If the intervals between DNCB sensitizations were sufficiently long, usually 7 days or longer, presumably for the animals to produce DNCB-specific Abs, then highly consistent and marked ear swelling could be readily observed. However, before DNCB-specific Abs became detectable, the magnitude of ear swelling in DNCB-treated animals was almost always under 50% regardless of the variations in other factors.

### Conclusion

The results of our present study demonstrate that the presence of DNCB-specific Abs plays an important role in the development of CHS. The appearance of DNCB-induced skin dermatitis matches in timing the appearance of DNCB-specific Abs in sera. Adoptive transfer of sera containing DNCB-specific Abs from DNCB-treated mice produces a much stronger hypersensitivity skin reaction than the adoptive transfer of lymph node cells from the same donors. Moreover, DNCB-induced CHS is markedly diminished in transgenic B cell-deficient mice that cannot produce DNCB-specific Abs. It was also observed that treatment of animals with DNCB polarizes Th cells into Th2 differentiation by increasing the production of Th2 cytokines (IL-4, IL-5 and IL-10) while decreasing the production of Th1 cytokines (IFN-γ and IL-2). Taken together, these data demonstrate that the production of DNCB-specific Abs by activated B cells plays an important role in the pathogenesis of chemically-induced CHS. These observations may open up new therapeutic targets for alleviating human CHS conditions.

## Materials and Methods

### Animal Experiments

#### Mice

All experimental protocols involving the use of live animals were approved by the Institutional Animal Care and Use Committee (IACUC) at the University of Kansas Medical Center (Kansas City, KS). The 6- to 8-week-old male and female BALB/c mice and 7-week-old female ICR mice were obtained from Harlan Laboratories (Houston, TX). The B cell-deficient mice (B6.129S2-Igh-6^tm1Cgn^/J) and their background control C57BL/6J mice were obtained from the Jackson Laboratory (ME, USA), and were raised in a sterile environment. After arrival, they were allowed to acclimatize for about a week before they were used in the experiment. The animals were housed under controlled conditions of temperature (22°C) and photoperiod (12 h light/12 h dark cycle), and the animals were allowed free access to food and water throughout the experiment.

#### DNCB-induced contact dermatitis

To induce contact dermatitis, the animals were sensitized multiple times with painting 100 µL of 2% DNCB in 200-proof ethanol (ETOH) onto the shaved back skin at different time intervals. Three to five days after the last DNCB sensitization of the back skin, the animals were challenged on their left ears with 20 µL of 2% DNCB in ETOH once or twice. The control animals were painted with ETOH alone. Twenty-four h later, the ear swelling was evaluated by measuring the difference between the right and left ears in their thickness (measured by using an engineer's electronic micrometer) and also their weight (based on measuring a small round piece cut-out by using a sharp clamp).

The mice in the dexamethasone (DEX)-treated group received i.m. injections of DEX at 10 mg/kg b.w. once every two days beginning one day before the sensitization with DNCB and the injections lasted until the end of the experiment.

#### Adoptive transfer of CHS

The donor BALB/c mice were first immunized with DNCB or vehicle for three or four times, as described above. Then DNCB or vehicle was painted on their left ears, after which ear thickness and DNCB-specific IgG in serum were measured. Positive donors (DNCB-treated) developed strong hypersensitivity skin reaction and high titers of serum DNCB-specific Abs, while the negative donors (vehicle-treated) did not have appreciable hypersensitivity reaction or detectable levels of DNCB-specific Abs. Twenty-four h after DNCB painting on the left ears, mice from both groups were euthanized. Single-cell suspensions were made from a pool of LNs from postauricle, axilla and groin of the donor mice. Cell density was adjusted to 5×10^8^ cells/mL in PBS for injection. From the same animals, fresh sera were also prepared. For the adoptive transfer, 100 µL of the lymphocyte suspension (5×10^7^ cells) or 300 µL of fresh serum or combination of lymphocyte suspension and serum from positive donors or negative donors was injected intravenously (i.v.) into individual recipient mouse in different groups. After that, all recipient animals were challenged on the left ears with 20 µL of 2% DNCB twice with a 60-min interval. After 18 and 66 h, ear swelling in the recipient mice was measured and serum DNCB-specific Ab levels were determined by using ELISA.

### Chemicals and Reagents

Dinitrochlorobenzene (DNCB, 99% purity) was purchased from Acros Organics (NJ, USA). Tween-20, albumin from chicken egg white (ovalbumin, 98% purity) and 1,2-phenylenediamine (OPD, 99.5% pure) were obtained from Sigma-Aldrich (MO,USA). Anti-mouse CD3-PE, anti-mouse CD4-FITC, anti-mouse CD8α-PE-Cy5, anti-mouse TCRβ-PE, and anti-mouse TCRγδ-PE were purchased from BD Biosciences (CA, USA). Anti-mouse CD45R (B220)-PE, anti-mouse F4/80-PE and purified anti-mouse IgA, IgM were obtained from eBioscience (CA, USA). HRP-conjugated anti-mouse IgA, IgG, IgG1, IgG2a, IgG2b, IgG3 and IgM were obtained from Santa Cruz Biotechnology (CA, USA), and the purified anti-mouse IgG, IgE as well as HRP-conjugated anti-mouse IgE were obtained from Chemicon (CA, USA).

The DNP-ovalbumin conjugate was prepared by dissolving 600 mg ovalbumin in 60 mL sodium borate buffer (0.05 M, pH 9.4). The solution was stirred at 4°C, and the DNCB powder (0.01 mol) was gradually added into the solution. The conjugate solution was stirred for 9 days at 4°C and then dialyzed against a sodium borate buffer for 16 days at 4°C. It is of note that while 9 days of conjugation reaction followed by 16 days of dialysis appeared to be very long, the outcome was much better than the procedures of a 2-day conjugation followed by a 2-day dialysis. The solution was centrifuged at 2000 *rpm* for 5 min. The supernatants were sequentially dialyzed against distilled water for 6 days at 4°C. Conjugates were then lyophilized and stored at −80°C until use. This protocol used in this study was modified according to an earlier study [34].

### Flow Cytometric Analysis

Immediately after the LNs (postauricular and inguinal lymph nodes), spleens and thymus were removed, they were grinded and the cells were strained to obtain the single cell suspensions. For the splenocytes, the red blood cells were lysed and washed before the following steps. Cell number was determined by using a hemocytometer. After incubation with the Ab conjugated with the fluorochrome(s) (FITC or PE or PE-CY5 or three in combination) followed by washing twice with PBS containing 2% FBS, the samples were fixed with 2% paraformaldehyde in PBS overnight and tested on a flow cytometer (Coulter EPICS XL-MCL 414102 from Beckman Coulter, Fullerton, CA). The data were analyzed using the Expo 32 ADC and FlowJo7 software.

### Histopathological and Immunohistochemical (IHC) Analyses

Formalin-fixed, paraffin-embedded ear or back skin tissues were sectioned at 5 µm in thickness, and the sections were stained with hematoxylin and eosin (H&E). The pictures were taken with a light microscope (magnification 100×).

IHC staining was performed according to the manufacturer's instructions with slight modifications. The ear and back skin tissues were collected from BALB/c mice sensitized and then challenged with DNCB or vehicle, and they were immediately frozen in dry-ice and kept at −80°C. The frozen tissues were then embedded in optimum cutting temperature compound (OTC) and cut into 6-µm thick sections and then air-dried at room temperature for 30 min. During staining, the slides were fixed in 2% (*v/v*) formaldehyde in PBS for 8 min at 4°C. After blocking the endogenous peroxidase activity and endogenous biotin, the sections were incubated overnight with a primary Ab (anti-mouse IFN-γ or anti-mouse IL-5) and then incubated with the biotinylated secondary Ab in a humidified chamber (all Abs were from R&D Systems, MN, USA). The slides were then incubated with Vectastain Elite ABC-peroxidase (Vector Laboratories, Burlingame, CA), then with 3, 3′-diaminobenzidine (Vector Laboratories, Burlingame, CA), and counterstained.

### Measurement of DNCB-Specific Ab Levels in Sera

After the peripheral blood was collected, it was kept at room temperatures for 1 h and then centrifuged at 3500 *rpm* (Eppendorf 5415r) at 4°C for 20 min, and the supernatants (sera) were stored at −80°C in aliquots until use. The serum Abs were tested by using ELISA, and all steps were carried out at room temperature.

For ELISA, the 96-well plates (obtained from NUNC) were first coated with 0.1 mg/mL DNP-ovalbumin conjugate in 0.1 M NaHCO_3_ overnight, and each well was then blocked with 0.25% gelatin in PBS for 1 h followed by washing with 0.05% Tween-20 in PBS (washing buffer). Properly diluted serum samples were then added into each well and incubated for 2–3 h. After 3 times of washing, the HRP-conjugated detection Ab was added. After incubation in the dark for 2 h and washing 3 times, the substrate solution (containing 1 mg/mL OPD and 1.5 µL/mL 30% hydrogen peroxide in 0.1 M citrate buffer, pH 5.0) was added into each well. After incubation in the dark for another 30 min, the plate was read on a Kinetic Microplate Reader (Molecule Devices) at OD_450 nm_ and OD_570 nm_. The final reading values were calculated using OD_450 nm_–OD_570 nm_.

### Assay of Primary Lymphocyte Proliferation and Measurement of Cytokine Release

LN cells and splenocytes were isolated from BALB/c mice after their left ears were challenged with DNCB or vehicle. Single-cell suspensions were prepared as described above. LN cells were seeded in 96-well plates at 5×10^5^ cells/well in 200 µL DMEM containing 10% (*v/v*) fetal bovine serum, while splenocytes were seeded in 96-well plate at 1×10^6^ cells/well in 200 µL medium. The cells were stimulated with 5 µg/mL Con A. After 22 and 41 h, an aliquot of the cell culture supernatant was collected and stored at −80°C for measurement of cytokine levels (described below). To determine the rate of cell proliferation, 200 µL media containing 2.5 µCi/mL [^3^H]thymidine was added into each well. After incubation for 14 h, cells were transferred onto the membrane A by using a cell harvester and read on a Beta scintillation counter (MicroBeta Trilux, PerkinElmer Life Sciences, MA, USA).

The levels of IL-2, IL-4, IL-10, IL-12 (p70) and IFN-γ released into the culture medium were measured by using the ELISA according to the manufacturer's instructions. Anti-mouse IL-4/IFN-γ (purified), recombinant mouse IL-4/IFN-γ and anti-mouse IL-4/IFN-γ-biotin were purchased from eBioscience (CA, USA) while the mouse IL-2, IL-10 and IL-12 (p70) cytokine ELISA kits were obtained from BD Biosciences (CA, USA).

### Statistical Analysis

All data are presented as mean±S.D. and were analyzed by paired and unpaired two-tailed Student's *t* tests. Difference between the groups was considered statistically significant when *P*<0.05.

## Supporting Information

Table S1 Comparison of ear swelling and the levels of DNCB-specific serum Abs in mice under different DNCB treatment conditions. In the experiments summarized below, the animal gender, DNCB dose, treatment time, solvent, site of sensitization and also other factors were changed to compare the degree of ear swelling and the levels of DNCB-specific serum Abs.
(0.07 MB DOC)

Figure S1 The contact hypersensitivity reactions seen at 24 h after painting 2% DNCB is not due to non-specific inflammatory reactions^1^. In one of the experiments shown above when 2% DNCB was used as a challenge in the absence of prior DNCB sensitization, we found that DNCB did not cause any significant ear swelling. The detailed experimental condition and results for this experiment are provided below. The mice as shown above in the left bar (in both panels) were sensitized with 2% DNCB by painting on their back skins on day 0 and challenged on their left ears on day 5. The mice as shown in the middle bar were painted with vehicle on their back skins on day 0 and challenged with vehicle on their left ears on day 5. The mice as shown in the right bar were only painted on their left ears with 2% DNCB. The ear swelling was measured at 24 h after DNCB ear challenge. The ear swelling indices were calculated according to the difference between the DNCB-challenged left ear and the unchallenged right ear using the following formula: index = the measured value for the left ear/the measured value for the right ear - 1. * P<0.01 compared to animals sensitized and challenged with DNCB. NS: no significance. Each value is the mean±S.D. (N = 4 for each group). ^1^FOOTNOTE: Consistent with the observation from the above experiment, it is also of note that when the animals received their first sensitization with 2% DNCB painted on their back skins, no visible inflammatory changes were seen in the first few days following the painting. This observation also showed that painting 2% DNCB alone did not cause significant non-specific inflammatory reactions at the site of topical application (in this case, the back skins) at early time points. Usually, relatively mild skin inflammatory changes could be seen from 8 days after DNCB painting (in the absence of DNCB challenge). Explanation for this late inflammatory reaction is provided in the main text.
(0.13 MB DOC)

Figure S2 Effect of DEX treatment on the body weight and organ weight indices in mice. In this experiment, the animals in the DNCB or DNCB + DEX group were painted on D0 and D12 with DNCB on their shaved back skins (as sensitizations), and on D17, the animals were challenged with painting DNCB on their left ears. Painting of ETOH (which served as the solvent) was used for the control group. All the animals in the DEX-treated group also received i.m. injection of DEX once every other day starting one day before the first DNCB sensitization. The body weight change of the animals during the experiment is shown in panel A, and the changes in the weight indices of spleen and thymus are shown in panel B. * P<0.01 compared to the animals treated with DNCB alone (N = 6 for each group).
(0.32 MB DOC)

Figure S3 Correlation of the severity of CHS with serum levels of DNCB-specific Abs. Serum levels of DNCB-specific Abs in mice treated with DNCB, Control, or DNCB + DEX were collected on D18 and assayed by using ELISA.
(0.18 MB DOC)

Figure S4 Effect of DNCB or DNCB + DEX treatment on the total serum levels of various Ig subtypes. Blood samples were taken on D18 following the first DNCB sensitization, and the total serum levels of IgA, IgM, IgG, IgG1, IgG2a, IgG2b and IgG3 were measured by using ELISA for all treatment groups (i.e., DNCB, control, and DNCB + DEX) in BALB/c mice. The ELISA assays were carried out in 96-well plates that were coated with respective capture Abs diluted in 0.1 M NaHCO_3_, and other procedures were the same as the measurement of the DNCB-specific Abs. N = 6 for each treatment group.
(0.24 MB DOC)

Figure S5 Changes in the cell populations (TCRβ^+^, CD3^+^CD4^+^, B220^+^ and IgG^+^) in both inguinal and postauricular lymph nodes following treatment with DNCB or vehicle. The DNCB treatment schedule was the same as described in Figure 1A. The data shown here were from one representative of four separate experiments which all showed a similar trend.
(0.52 MB DOC)

## References

[1] Garrigue JL, Nicolas JF, Fraginals R, Benezra C, Bour H (1994). Optimization of the mouse ear swelling test for in vivo and in vitro studies of weak contact sensitizers.. Contact Dermatitis.

[2] Watanabe H, Unger M, Tuvel B, Wang B, Sauder DN (2002). Contact hypersensitivity: the mechanism of immune responses and T cell balance.. J Interferon Cytokine Res.

[3] Grabbe S, Schwarz T (1998). Immunoregulatory mechanisms involved in elicitation of allergic contact hypersensitivity.. Immunol Today.

[4] Tuckermann JP, Kleiman A, Moriggl R, Spanbroek R, Neumann A (2007). Macrophages and neutrophils are the targets for immune suppression by glucocorticoids in contact allergy.. J Clin Invest.

[5] Kehren J, Desvignes C, Krasteva M, Ducluzeau MT, Assossou O (1999). Cytotoxicity is mandatory for CD8(+) T cell-mediated contact hypersensitivity.. J Exp Med.

[6] Xu H, Banerjee A, Dilulio NA, Fairchild RL (1997). Development of effector CD8+ T cells in contact hypersensitivity occurs independently of CD4+ T cells.. J Immunol.

[7] Wang B, Fujisawa H, Zhuang L, Freed I, Howell BG (2000). CD4+ Th1 and CD8+ type 1 cytotoxic T cells both play a crucial role in the full development of contact hypersensitivity.. J Immunol.

[8] Larsen JM, Geisler C, Nielsen MW, Boding L, Von Essen M (2007). Cellular dynamics in the draining lymph nodes during sensitization and elicitation phases of contact hypersensitivity.. Contact Dermatitis.

[9] Watanabe R, Fujimoto M, Ishiura N, Kuwano Y, Nakashima H (2007). CD19 expression in B cells is important for suppression of contact hypersensitivity.. Am J Pathol.

[10] Askenase PW (2001). Yes T cells, but three different T cells (alphabeta, gammadelta and NK T cells), and also B-1 cells mediate contact sensitivity.. Clin Exp Immunol.

[11] Campos RA, Szczepanik M, Itakura A, Lisbonne M, Dey N (2006). Interleukin-4-dependent innate collaboration between iNKT cells and B-1 B cells controls adaptative contact sensitivity.. Immunology.

[12] Fei M, Wu X, Xu Q (2005). Astilbin inhibits contact hypersensitivity through negative cytokine regulation distinct from cyclosporin A.. J Allergy Clin Immunol.

[13] O'Leary JG, Goodarzi M, Drayton DL, von Andrian UH (2006). T cell- and B cell-independent adaptive immunity mediated by natural killer cells.. Nat Immunol.

[14] Perone MJ, Bertera S, Shufesky WJ, Divito SJ, Montecalvo A (2009). Suppression of autoimmune diabetes by soluble galectin-1.. J Immunol.

[15] Mills CD, Kincaid K, Alt JM, Heilman MJ, Hill AM (2000). M-1/M-2 macrophages and the Th1/Th2 paradigm.. J Immunol.

[16] Hsieh CS, Macatonia SE, O'Garra A, Murphy KM (1995). T cell genetic background determines default T helper phenotype development in vitro.. J Exp Med.

[17] Wang B, Feliciani C, Freed I, Cai Q, Sauder DN (2001). Insights into molecular mechanisms of contact hypersensitivity gained from gene knockout studies.. J Leukoc Biol.

[18] Traidl C, Jugert F, Krieg T, Merk H, Hunzelmann N (1999). Inhibition of allergic contact dermatitis to DNCB but not to oxazolone in interleukin-4-deficient mice.. J Invest Dermatol.

[19] Leung DY (1995). Atopic dermatitis: the skin as a window into the pathogenesis of chronic allergic diseases.. J Allergy Clin Immunol.

[20] Nagai J, Yano I, Hashimoto Y, Takano M, Inui K (1998). Efflux of intracellular alpha-ketoglutarate via p-aminohippurate/dicarboxylate exchange in OK kidney epithelial cells.. J Pharmacol Exp Ther.

[21] Nagai H, Ueda Y, Tanaka H, Hirano Y, Nakamura N (1999). Effect of overproduction of interleukin 5 on dinitrofluorobenzene-induced allergic cutaneous response in mice.. J Pharmacol Exp Ther.

[22] Weigmann B, Schwing J, Huber H, Ross R, Mossmann H (1997). Diminished contact hypersensitivity response in IL-4 deficient mice at a late phase of the elicitation reaction.. Scand J Immunol.

[23] Yokozeki H, Ghoreishi M, Takagawa S, Takayama K, Satoh T (2000). Signal transducer and activator of transcription 6 is essential in the induction of contact hypersensitivity.. J Exp Med.

[24] Thomson JA, Troutt AB, Kelso A (1993). Contact sensitization to oxazolone: involvement of both interferon-gamma and interleukin-4 in oxazolone-specific Ig and T-cell responses.. Immunology.

[25] Stevens TL, Bossie A, Sanders VM, Fernandez-Botran R, Coffman RL (1988). Regulation of antibody isotype secretion by subsets of antigen-specific helper T cells.. Nature.

[26] Miles SA, Conrad SM, Alves RG, Jeronimo SM, Mosser DM (2005). A role for IgG immune complexes during infection with the intracellular pathogen Leishmania.. J Exp Med.

[27] Cohen S, Ward PA (1971). In vitro and in vivo activity of a lymphocyte and immune complex-dependent chemotactic factor for eosinophils.. J Exp Med.

[28] Yamada N, Wakugawa M, Kuwata S, Yoshida T, Nakagawa H (1995). Chronologic analysis of in situ cytokine expression in mite allergen-induced dermatitis in atopic subjects.. J Allergy Clin Immunol.

[29] Wang LF, Sun CC, Wu JT, Lin RH (1999). Epicutaneous administration of hapten through patch application augments TH2 responses which can downregulate the elicitation of murine contact hypersensitivity.. Clin Exp Allergy.

[30] Nakazawa M, Sugi N, Kawaguchi H, Ishii N, Nakajima H (1997). Predominance of type 2 cytokine-producing CD4+ and CD8+ cells in patients with atopic dermatitis.. J Allergy Clin Immunol.

[31] Shaala AY, Hodgson C, Ling NR, Jefferis R (1980). B lymphocytes in contact dermatitis.. Br J Dermatol.

[32] Carapeto FJ, Winkelmann RK, Jordon RE (1976). T and B lymphocytes in contact and atopic dermatitis.. Arch Dermatol.

[33] Kitagaki H, Ono N, Hayakawa K, Kitazawa T, Watanabe K (1997). Repeated elicitation of contact hypersensitivity induces a shift in cutaneous cytokine milieu from a T helper cell type 1 to a T helper cell type 2 profile.. J Immunol.

[34] Warbrick EV, Dearman RJ, Kimber I (2002). Induced changes in total serum IgE concentration in the Brown Norway rat: potential for identification of chemical respiratory allergens.. J Appl Toxicol.

