# Supplementary material for: Mechanism of Dinitrochlorobenzene-Induced Dermatitis in Mice: Role of Specific Antibodies in Pathogenesis
Source: PLoS One. 2009 Nov 5;4(11):e7703. doi: 10.1371/journal.pone.0007703 (PMC2766640; doi:10.1371/journal.pone.0007703)
Supplement: Table S1 — Comparison of ear swelling and the levels of DNCB-specific serum Abs in mice under different DNCB treatment conditions. In the experiments summarized below, the animal gender, DNCB dose, treatment time, solvent, site of sensitization and also other factors were changed to compare the degree of ear swelling and the levels of DNCB-specific serum Abs. (0.07 MB DOC) [file pone.0007703.s001.doc]

**Supplementary Table 1**

|  | **sex** | **Dose** | | **time (day)** | | **vehicle** | | **sensitization site** | **Δ ear thickness** | | **Δ ear weight** | | **DNCB-specific Abs** |
| --- | --- | --- | --- | --- | --- | --- | --- | --- | --- | --- | --- | --- | --- |
|  |  | **sensitization** | **challenge** | **sensitization** | **challenge** | **sensitization** | **challenge** |  | **DNCB** | **vehicle** | **DNCB** | **vehicle** |  |
| Exp-1 | *f* | 100 μL 2% | 30 μL 2% | 0, 4 | 5 | ETOH | ETOH | abdomen | 0.524 | 0.05 | 0.427 | -0.225 | no |
| Exp-2 | *m* | 50 μL 1% | 25 μL 1% | 0, 1 ,2 | 7, 11 | ETOH | corn oil | back | 0.253  ± 0.012 | 0.013  ± 0.041 | 0.433 ± 0.014 | 0.006  ± 0.006 | no |
| Exp-3 | *m* | 50 μL 2% | 30 μL 2% | 0 | 6 | ETOH | corn oil | back | 0.201  ± 0.025 | 0.000  ± 0.006 | 0.276  ± 0.097 | 0.079  ± 0.157 | no |
| Exp-4 | *f* | 100 μL 1% | 30 μL 1% | 0 | 3, 4 | ETOH | corn oil | back | 0.219 | 0.000  ± 0.009 | 0.540 | 0.073  ± 0.091 | no |
|  |  |  |  | 0, 1 | 5 |  |  |  | 0.0872  ± 0.011 |  | 0.308  ± 0.128 |  | no |
|  |  |  |  | 0 | 5 |  |  |  | 0.060  ± 0.032 |  | 0.274  ± 0.083 |  | no |
|  |  | 100 μL 0.5% | 30 μL 0.5% |  |  |  |  |  | 0.0755  ± 0.010 |  | 0.281  ± 0.174 |  | no |
|  |  | 100 μL 2% | 30 μL 2% |  |  |  |  |  | 0.191  ± 0.054 |  | 0.499  ± 0.098 |  | no |
| Exp-5 | *m* | 100 μL 1% | 30 μL 1% | 0 | 5 | ETOH | corn oil | back | 0.019 | 0.002 | 0.336 | 0.010 | no |
|  |  |  |  |  |  |  |  | abdomen | 0.092 |  | 0.104 |  | no |
|  | *f* |  |  |  |  |  |  | back | 0.050 | -0.002 | 0.176 | -0.007 | no |
|  |  |  |  |  |  |  |  | abdomen | 0.087 |  | 0.200 |  | no |
| Exp-6 | *m* | 50 μL 0.1% | 30 μL 1% | 0 | 4 | corn oil | ETOH | s.c. injection | 0.245 | 0.009 | 0.257 | 0.023 | no |
|  |  | 50 μL 0.05 |  |  |  |  |  |  | 0.356 |  | 0.411 |  | no |
|  |  | 50 μL 0.02 |  |  |  |  |  |  | 0.216 |  | 0.542 |  | no |
|  |  | 100 μL 1% |  |  |  | ETOH |  |  | 0.289 |  | 0.465 |  | no |
| Exp-7 | *f* | 100 μL 1% | 20 μL 2% × 2 | 0 | 4 | ETOH | ETOH | back | 0.246 | 0.008 | 0.440 | -0.073 | no |
| Exp-8 | *f* | 100 μL 2% | 20 μL 2% × 2 | 0 | 3. 5 | ETOH | ETOH | back | 0.431  ± 0.072 | 0.003  ± 0.018 | 0.454  ± 0.168 | -0.010  ± 0.027 | no |
| Exp-9  (ICR mice) | *f* | 100 μL 2% | 20 μL 2% × 2 | 0 | 3. 5 | ETOH | ETOH | back | 0.427  ± 0.046 | -0.006  ± 0.027 | 0.600  ± 0.091 | 0.029  ± 0.063 | no |
| Exp-10 | *f* | 100 μL 2% | 20 μL 2% × 2 | 0, 12 | 17 | ETOH | ETOH | back | 0.818  ± 0.038 | -0.003  ± 0.011 | 0.968  ± 0.096 | -0.017  ± 0.028 | yes |
| Exp-11 | *f* | 100 μL 2% | 20 μL 2% × 2 | 0, 7, 13 | 17 | ETOH | ETOH | back | 1.132  ± 0.128 | 0.002  ± 0.011 | 1.867  ± 0.580 | 0.035 ± 0.026 | yes |
