# Supplementary figures and images for: Mechanism of Dinitrochlorobenzene-Induced Dermatitis in Mice: Role of Specific Antibodies in Pathogenesis
Source: PLoS One. 2009 Nov 5;4(11):e7703. doi: 10.1371/journal.pone.0007703 (PMC2766640; doi:10.1371/journal.pone.0007703)

**Supplementary Figure 1**


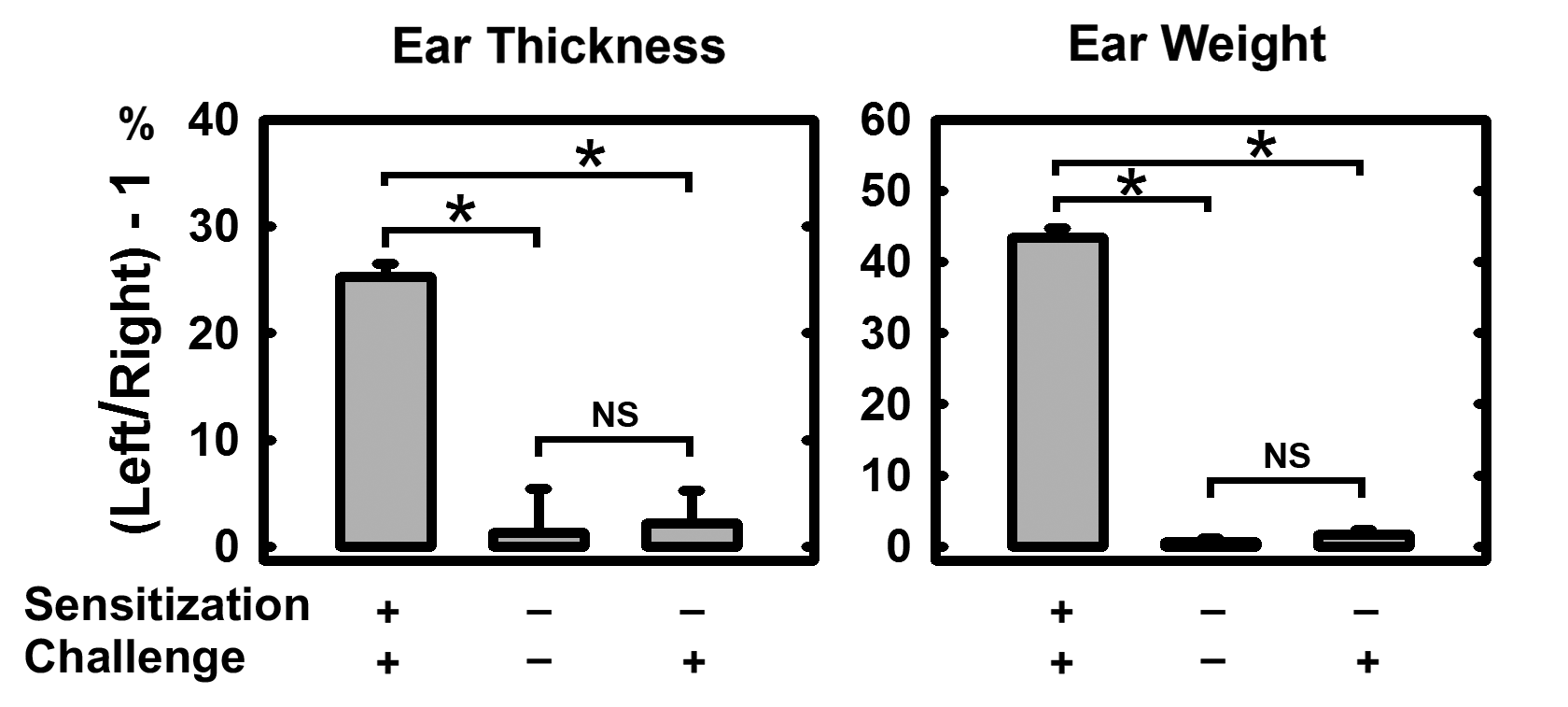

Supplement: Figure S1 — The contact hypersensitivity reactions seen at 24 h after painting 2% DNCB is not due to non-specific inflammatory reactions1. In one of the experiments shown above when 2% DNCB was used as a challenge in the absence of prior DNCB sensitization, we found that DNCB did not cause any significant ear swelling. The detailed experimental condition and results for this experiment are provided below. The mice as shown above in the left bar (in both panels) were sensitized with 2% DNCB by painting on their back skins on day 0 and challenged on their left ears on day 5. The mice as shown in the middle bar were painted with vehicle on their back skins on day 0 and challenged with vehicle on their left ears on day 5. The mice as shown in the right bar were only painted on their left ears with 2% DNCB. The ear swelling was measured at 24 h after DNCB ear challenge. The ear swelling indices were calculated according to the difference between the DNCB-challenged left ear and the unchallenged right ear using the following formula: index = the measured value for the left ear/the measured value for the right ear - 1. * P<0.01 compared to animals sensitized and challenged with DNCB. NS: no significance. Each value is the mean±S.D. (N = 4 for each group). 1FOOTNOTE: Consistent with the observation from the above experiment, it is also of note that when the animals received their first sensitization with 2% DNCB painted on their back skins, no visible inflammatory changes were seen in the first few days following the painting. This observation also showed that painting 2% DNCB alone did not cause significant non-specific inflammatory reactions at the site of topical application (in this case, the back skins) at early time points. Usually, relatively mild skin inflammatory changes could be seen from 8 days after DNCB painting (in the absence of DNCB challenge). Explanation for this late inflammatory reaction is provided in the main text. (0.13 MB DOC) [file pone.0007703.s002.doc]

**Supplementary Figure 2**


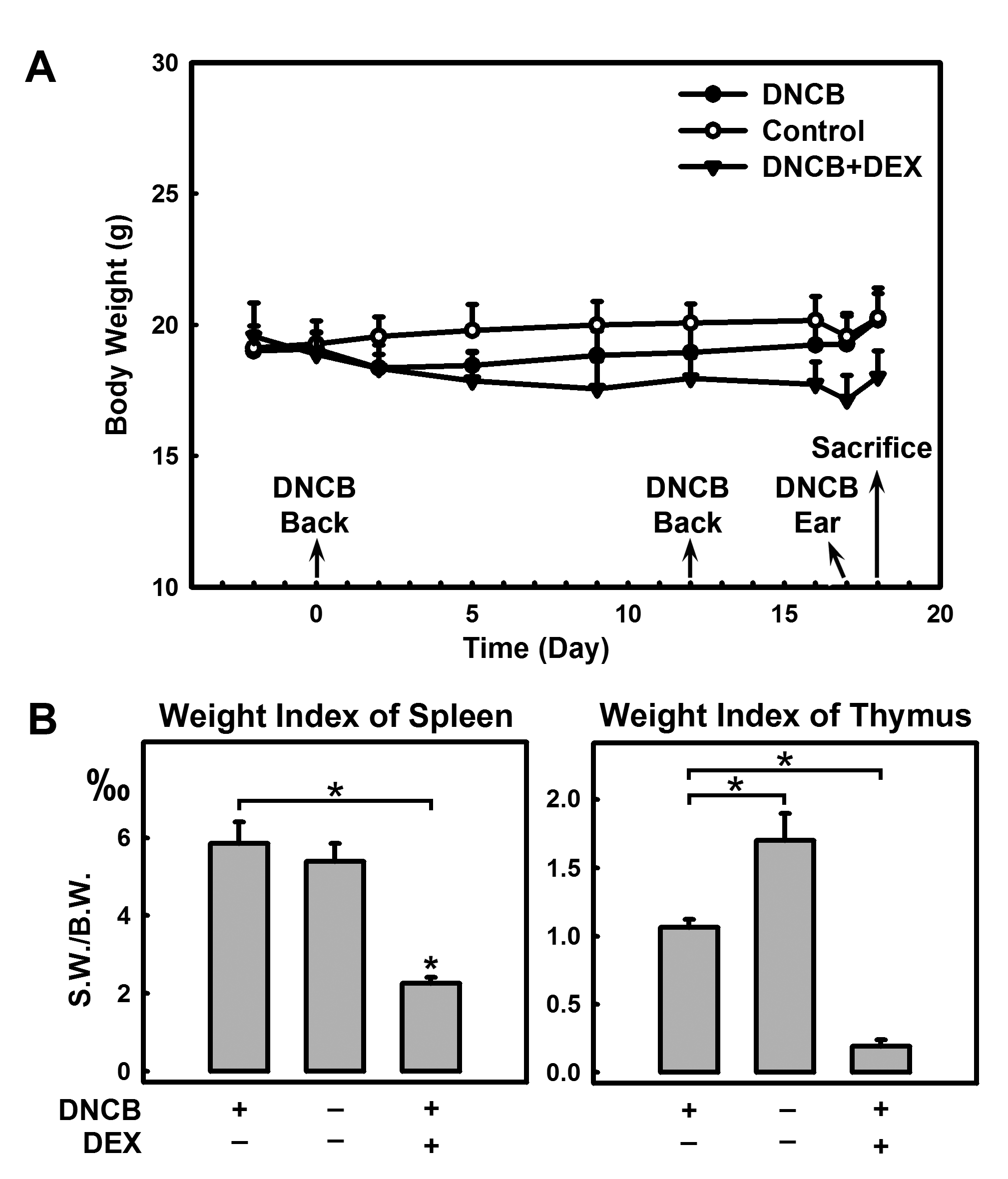

Supplement: Figure S2 — Effect of DEX treatment on the body weight and organ weight indices in mice. In this experiment, the animals in the DNCB or DNCB + DEX group were painted on D0 and D12 with DNCB on their shaved back skins (as sensitizations), and on D17, the animals were challenged with painting DNCB on their left ears. Painting of ETOH (which served as the solvent) was used for the control group. All the animals in the DEX-treated group also received i.m. injection of DEX once every other day starting one day before the first DNCB sensitization. The body weight change of the animals during the experiment is shown in panel A, and the changes in the weight indices of spleen and thymus are shown in panel B. * P<0.01 compared to the animals treated with DNCB alone (N = 6 for each group). (0.32 MB DOC) [file pone.0007703.s003.doc]

**Supplementary Figure 3**


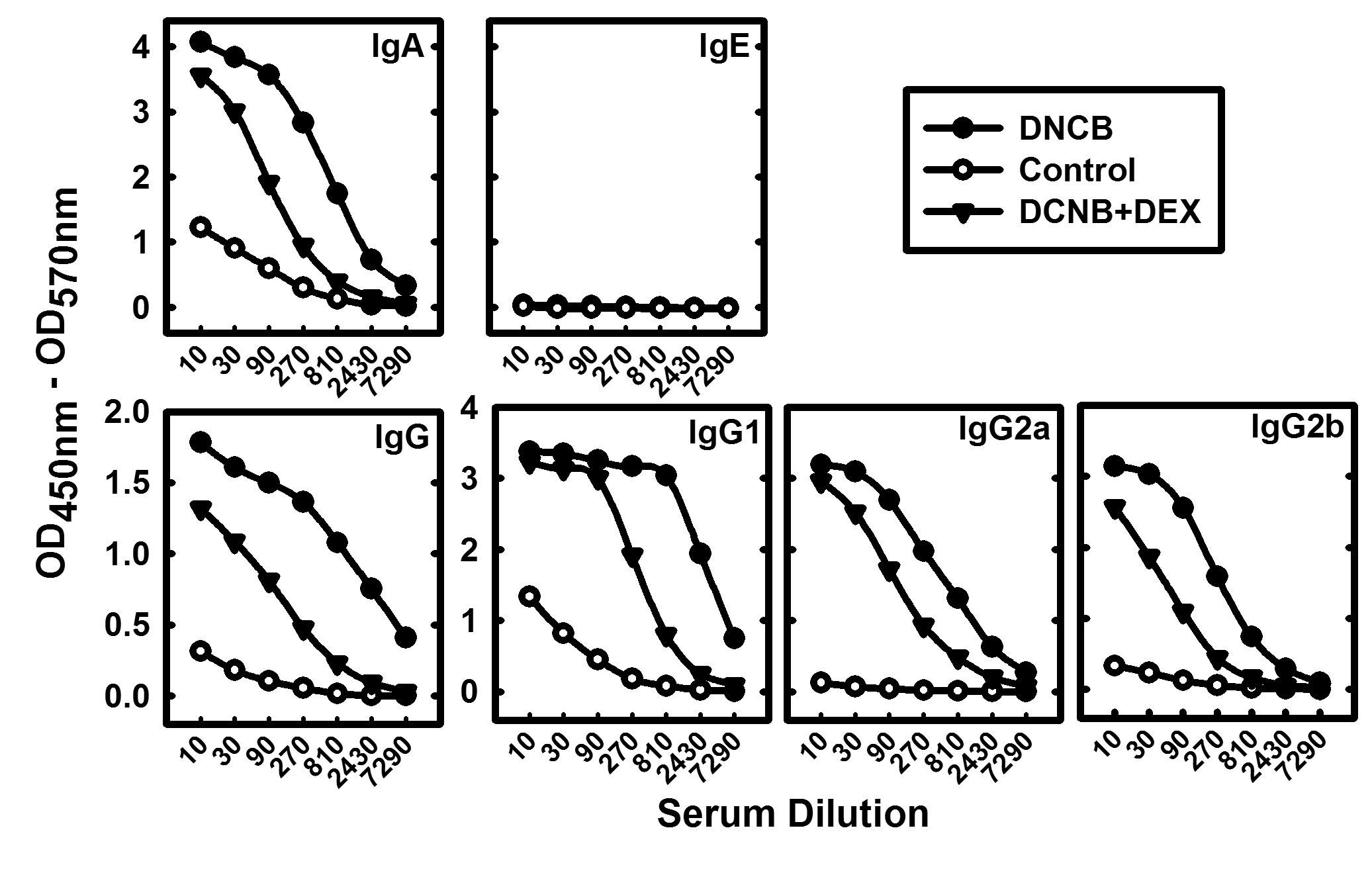

Supplement: Figure S3 — Correlation of the severity of CHS with serum levels of DNCB-specific Abs. Serum levels of DNCB-specific Abs in mice treated with DNCB, Control, or DNCB + DEX were collected on D18 and assayed by using ELISA. (0.18 MB DOC) [file pone.0007703.s004.doc]

**Supplementary Figure 4**


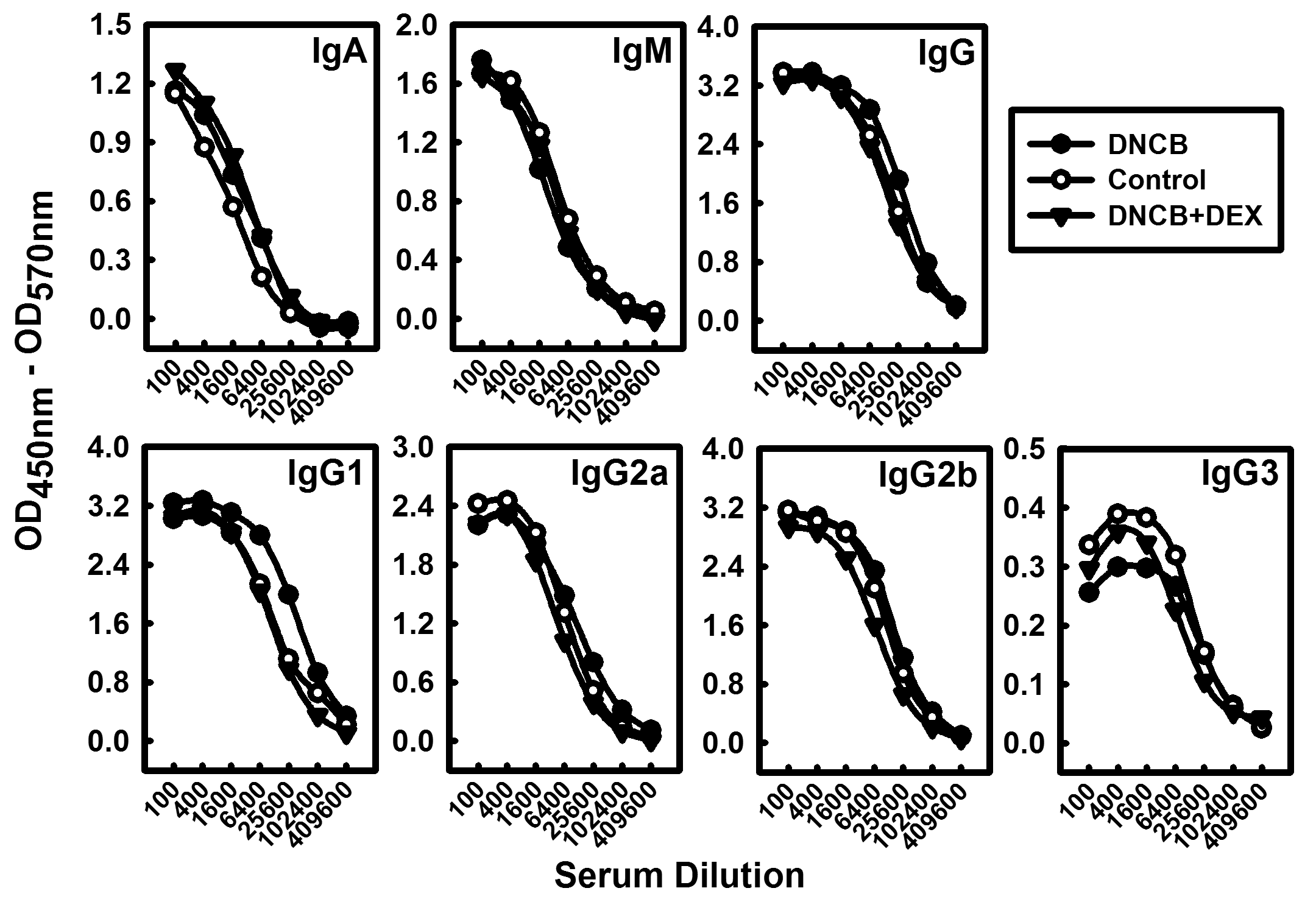

Supplement: Figure S4 — Effect of DNCB or DNCB + DEX treatment on the total serum levels of various Ig subtypes. Blood samples were taken on D18 following the first DNCB sensitization, and the total serum levels of IgA, IgM, IgG, IgG1, IgG2a, IgG2b and IgG3 were measured by using ELISA for all treatment groups (i.e., DNCB, control, and DNCB + DEX) in BALB/c mice. The ELISA assays were carried out in 96-well plates that were coated with respective capture Abs diluted in 0.1 M NaHCO3, and other procedures were the same as the measurement of the DNCB-specific Abs. N = 6 for each treatment group. (0.24 MB DOC) [file pone.0007703.s005.doc]

**Supplementary Figure 5**


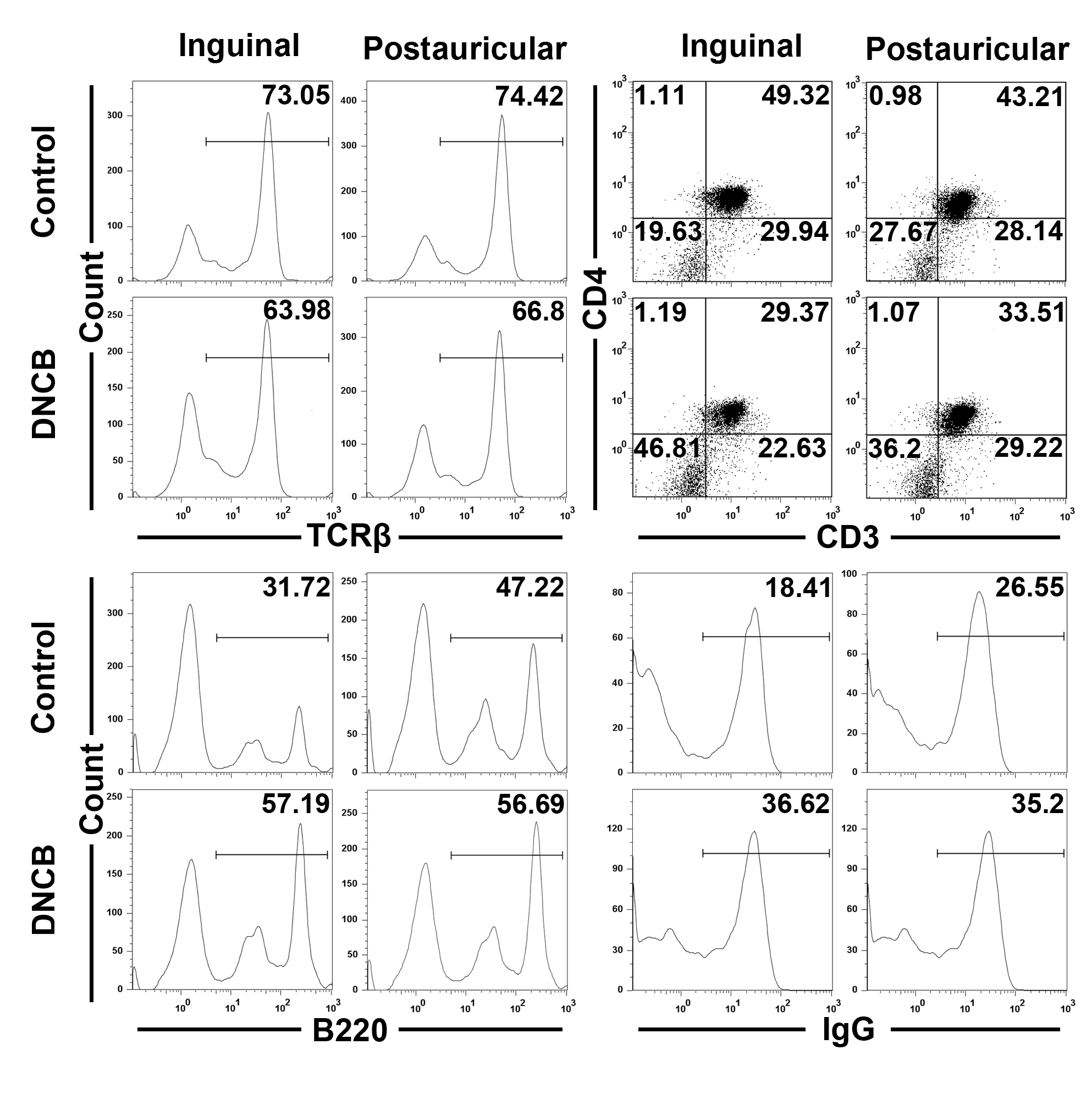

Supplement: Figure S5 — Changes in the cell populations (TCRβ+, CD3+CD4+, B220+ and IgG+) in both inguinal and postauricular lymph nodes following treatment with DNCB or vehicle. The DNCB treatment schedule was the same as described in Figure 1A. The data shown here were from one representative of four separate experiments which all showed a similar trend. (0.52 MB DOC) [file pone.0007703.s006.doc]
